# Beyond human limits: the ethical, social, and regulatory implications of human enhancement

**DOI:** 10.3389/fmed.2025.1595213

**Published:** 2025-07-09

**Authors:** Chiara Gerardi, Christodoulos Xinaris

**Affiliations:** Istituto di Ricerche Farmacologiche "Mario Negri" IRCCS, Milan, Italy

**Keywords:** human enhancement, gene editing, regulatory, ethics, cognitive enhancement, physical enhancement

## Abstract

Human enhancement involves interventions that enhance physical or cognitive performance or overall wellbeing beyond typical human limits. The consequence of human enhancement has the potential to radically reshape society and the very existence of our species, requiring careful consideration of its ethical and regulatory boundaries. In this paper, we explore current applications of human enhancement, including motor neuro -prostheses, transcranial magnetic stimulation, and gene therapy. We analysed key unresolved challenges and potential solutions, focusing on regulatory and policy frameworks, ethical considerations in clinical research, transparency through registries, traceability of interventions, and the role of informed decision-making and consent. Finally, we critically examine the evolving definition of health and the need to delineate clear boundaries between prevention and enhancement. By addressing these issues, this paper aims to contribute to the ongoing dialog on the responsible development and implementation of human enhancement technologies.

## Introduction

Human enhancement refers to the application of biomedical, biotechnological, and other scientific interventions to improve physical, cognitive, emotional, or overall wellbeing beyond what is considered typical or necessary for health (1). While some forms of human enhancement address health conditions, others seek to reduce risk of predictable diseases and/or to enhance the abilities of healthy individuals, raising important ethical questions about the societal implications of these interventions. These debates, which encompass ontological, biological, and social aspects, focus on the methods used to improve the physical, cognitive, or emotional status of individuals, as well as on the very definition of what it means to be human.

Humanity has always tried to go beyond natural limits, making human enhancement not only part of today’s cultural zeitgeist, but a recurring theme in earliest myths and stories. For thousands of years people have tried to enhance their physical and cognitive capabilities—sometimes successfully and other times with questionable or catastrophic results.

One of the most enduring examples in human history is Icarus’ legend. Exhilarated by the power of his artificial wings, ignored his father’s warnings and flew too close to the sun. In doing so, he exceeded the natural limits and moderation—what the Greeks referred to as *metron*—causing his wax-bound wings to melt, and he fell into the Aegean Sea. While Daedalus’ scientific principle was visionary and valid, its reckless application led to disastrous consequences.

Throughout history, humanity has witnessed many “Icarus effects”—instances where scientifically sound advancements have been taken to dangerous extremes, often resulting in catastrophic and unethical outcomes. From the misuse of nuclear energy in warfare to unethical medical experiments like the Tuskegee Study (2), the pattern remains clear: when technological ambition surpasses ethical foresight, progress can turn into peril.

The scope and potential consequences of human enhancement demand an in-depth discussion regarding the societal acceptance of such interventions and the boundaries that must be established for their ethical use. The rapid advancements of fields such as AI and bioengineering, gene therapies pose substantial challenges for policymakers (3). Designing a regulatory framework that ensures safety, equity and ethical oversight of these innovations is an urgent need. This is particularly imperative in the contemporary landscape, where technological innovation and its global dissemination occur at unprecedented speeds and can potentially outpace ethical and regulatory safeguards.

In this viewpoint, we explored the current applications of human enhancement, the unresolved challenges, and potential solutions. We focused also our attention on equity, access, and the risk of social stratification related to the human enhancement. We finally analysed ethical considerations, regulatory and policy frameworks, research practices, traceability, and the critical importance of informed decision-making and consent.

## Human enhancement and current applications

Human enhancement can be cognitive, physical, moral, or related to longevity. This paper focuses on cognitive and physical enhancement, providing some examples of these applications.

### Cognitive enhancement

Cognitive enhancement refers to the application of technologies and methods aimed at improving mental processes such as memory, attention, and executive functions in both healthy individuals and those with cognitive impairments (4).

One of the most recent (and extensively discussed) innovations in this field is Neuralink, a brain-computer interface (BCI) company. Neuralink’s technologies are designed to enable direct communication between the brain and computers, potentially allowing for enhancement of cognitive capabilities. The primary application of this technology is to address critical problems associated with conditions such as Alzheimer’s, spinal cord injuries, and other neurological disorders, while also opening the possibility for cognitive augmentation in healthy users by directly interfacing with external technologies (5).

While Neuralink’s technology is still in experimental stages, it exemplifies the broader category of invasive BCIs aimed at augmenting human cognition. For example, in August 2020, the Food and Drug Administration (FDA) granted Stentrode, a motor neuroprosthesis, developed by the company Synchron, with the Breakthrough Device designation, which is for medical devices that have the potential to provide improved treatment for debilitating or life-threatening conditions (6). They conducted a monocenter first-in-human study to assess the safety of an endovascular device incorporating recording electrodes and implanted in the superior sagittal sinus. This trial included five patients with severe bilateral upper-limb paralysis, due to amyotrophic lateral sclerosis in four participants and primary lateral sclerosis in one. They completed the follow-up with no serious adverse events and no vessel occlusion or device migration. This is another BCI implant that required a minimally invasive endovascular technique to deliver recording electrodes through the jugular vein to superior sagittal sinus, whereas other approaches must implant their BCIs through open-brain surgery (7).

Among the non-invasive technologies, transcranial magnetic stimulation (TMS) and transcranial direct current stimulation (tDCS) are two FDA-approved techniques for cognitive enhancement (8). TMS uses magnetic fields to stimulate nerve cells and is primarily applied to treat depression. However, it has shown potential in improving working memory and attention in both clinical and non-clinical populations (9). Likewise, tDCS applies mild electrical currents to modulate neural activity and has been tested for its ability to enhance problem-solving, learning, and attention (10). These examples highlight how cognitive enhancement technologies are being used to both treat mental health conditions and improve cognitive abilities in the general population.

### Gene therapy

Beyond cognitive technologies, gene therapy represents a transformative approach to both treating and modifying human traits, that falls on physical enhancement. Gene therapy is a medical approach designed to treat, prevent or cure a disease or medical disorder by replacing or correcting the underlying genetic defects (11). Both inherited genetic diseases (e.g., hemophilia and sickle cell disease) and acquired disorders (e.g., leukemia) could be treated with this medical approach (12). For instance, in the last years, the FDA and the European Medicines Agency (EMA) approved several gene therapies, such as voretigene neparvovec (called Luxturna) for treating retinal dystrophy (13, 14) and onasemnogene abeparvovec (called Zolgensma) for spinal muscular atrophy (15, 16) adding a new therapeutic chance for patients with a poor prognosis (17).

Beyond therapeutic applications, gene editing technologies like CRISPR-Cas9 have opened the door to enhancing human abilities. CRISPR allows precise modifications of DNA sequences, enabling scientists to potentially enhance traits such as muscle strength, disease resistance, or cognitive capabilities (18). In theory, CRISPR could be used to optimize intelligence, memory, and even emotional resilience, though such applications raise significant ethical questions (19). Box 1 reports a snapshot of clinical research on gene editing with CRISPR-Cas9 to illustrate clinical research ongoing on this topic.

BOX 1 **A snapshot of ongoing clinical research on gene editing with CRISPR-Cas9**

In October 2024, we looked at one of the main clinical trials registries such as Clinicaltrials.gov, to observe and report ongoing studies on gene editing and CRISPR-CAS9. Focusing our attention on ongoing clinical research on human enhancement, we found 16 studies aimed to assess the efficacy and safety of CRISPR-Cas9.

The aims of these studies are different:

- to treat oncological diseases CRISPR/Cas9 Instantaneous Gene Editing Therapy to Intraocular Hypertensive POAG With MYOC Mutation, prevention of sickle cell disease,
- to assess the safety and efficacy of CRISPR/Cas9 mRNA Instantaneous Gene Editing Therapy to treat refractory viral keratitis,
- to understand the efficacy of gene editing as a therapeutic approach for Rett Syndrome (MECPer-3D),
- Exploiting Epigenome Editing in Kabuki syndrome as a new route toward gene therapy for rare genetic disorders (Epi-KAB),
- to evaluate a first-in-class allogeneic anti-CD19 chimeric antigen receptor T-cell therapy UCART019 in patients with relapsed or refractory CD19 + Leukemia and Lymphoma (https://clinicaltrials.gov/search?cond=Gene%20Therapy&term=CRISPR-Cas9&page=1).

A striking example of gene therapy’s controversial potential emerged in 2018, when Chinese scientist He Jiankui genetically modified human embryos using CRISPR to make them resistant to HIV. He altered the CCR5 gene, which is linked to HIV resistance, but this modification may have also (possibly unintentionally) enhanced the intelligence of the children born from these embryos. CCR5 has been associated with cognitive functions like memory and learning in mice, raising ethical concerns about the long-term consequences and societal implications of such genetic modifications (20). This case highlights the fine boundaries between therapeutic use and enhancement, as well as the profound ethical challenges gene therapy presents.

At this point, two fundamental questions emerge: Should gene therapy be used not only to treat diseases but also to prevent them? And if so, what are the biological and ethical boundaries of what is considered a “disease”? These questions are critical in the context of preventable conditions such as obesity and cardiovascular disease (CVD). For instance, if genetic interventions could reduce the risk of developing obesity or heart disease, does that justify their use? Or would such interventions cross into the field of enhancement, where we are not just preventing illness but actively altering human biology to optimize certain traits?

## Equity, access, and social stratification

A critical ethical concern surrounding human enhancement technologies is their potential to exacerbate social inequality. If enhancements, whether genetic, cognitive, or physical—become widely available, will they be accessible to all, or only to those who can afford them? This question is particularly pressing given the current disparities in access to healthcare and medical services globally. If access to enhancements is limited by socioeconomic status, we risk creating a new form of social stratification, where individuals who are genetically or cognitively enhanced hold significant advantages in health, intelligence, and physical abilities.

Historically (from the agriculture revolution to the present day), social status has been based on wealth and resources, but selective genetic improvements could lead to the formation of biological castes, where one’s genetic identity becomes a new marker of privilege or disadvantage. This scenario poses a grave threat to social cohesion and even to the sustainability of our species. If some individuals are biologically engineered to possess superior traits—such as higher intelligence, increased strength, or disease resistance—it could widen existing social divides, creating a class of “genetically elite” who dominate others not just economically, but biologically.

Furthermore, germline editing, could have long-lasting societal consequences. As these modifications are passed down through generations, we may see the emergence of classes of individuals defined by the quality of their engineered genome. More critically, such interventions may limit evolutionary adaptability and reduce the genetic diversity necessary for resilience against environmental changes, emerging diseases and overall unpredictable future challenges. This possibility raises concerns about long-term societal impacts, as the creation of biologically distinct classes could perpetuate inequality across generations, threatening both the ethical foundations of equal opportunity and the broader stability of human society.

## Redefining disease and health

The line between therapy and enhancement is becoming increasingly blurred, especially for conditions that have both a biological basis and a strong environmental component. If we take again obesity, as an example, it is influenced not only by genetics but also by factors such as lifestyle, diet, and socioeconomic conditions (21). This raises a debate among two different positions: one is to adopt the enhancement to intervene at our genes to reduce the predisposition to obesity and the other one concerns a different approach addressing societal factors like education, access to healthcare, and promoting healthier living environments. If society and governments accepted this approach (to adopt genetic treatment for preventable diseases), we should define the boundaries of an acceptable use of the enhancement as well as the sustainability of this intervention. Where does prevention end and enhancement begin?

The distinction becomes even more complex when considering how society now perceives conditions like aging. Increasingly, aging itself is viewed as a treatable condition rather than a natural process, and diseases traditionally associated with aging—such as obesity, diabetes, and even resistance to infections—could be seen as targets for therapeutic enhancement. If interventions to slow aging or enhance resistance to diseases are classified as treatments, how do we differentiate them from enhancements aimed at optimizing health beyond what is considered “normal”?

Before (re)defining the limits of disease and health, a set of open issues should be addressed on the main points of discussion on human enhancement. To do that, we analyzed these key points with particular attention to the potential solutions and current strategies being used to tackle these challenges.

## Open issues and possible solutions

### Regulation and policy

Developing responsible legal frameworks for emerging technologies is an urgent need to be addressed.

Governments, with the involvement of citizens, must proactively establish regulatory frameworks considering the rapid evolving of human enhancement technologies. One solution could be a special committee or individual teams from various institutions dedicated to continually update legislation based on technological advancements. These efforts should be focused on developing clear regulatory guidelines on the safe and ethical use of genetic modification, BCIs, and AI-driven enhancements. It must ensure equitable access to enhancement technologies to prevent social inequalities and collaborate with international bodies to establish cross-border regulatory standards, particularly for gene editing and cognitive enhancement technologies. Regulation should also the aim to protect vulnerable populations including older adults, individuals with life-threatening diseases, children, marginalized communities, those with lower levels of education, and individuals from low-income countries.

### Ethical considerations and clinical research: increase global ethical standards on human enhancement

In 2021, the European Commission (EC) endorsed a set of ethics guidelines for research on human enhancement that is now included in the ethics review guidance for the Horizon Europe funding program (22, 23). The need arose from the observation that despite intense discussions in society and academia—few proposals demonstrated to be successful in establishing ethical guidance for the use and development of implants, drugs and prosthetics used to enhance human abilities.

This document is one of the main outputs of the SIENNA (Stakeholder-Informed Ethics for New technologies with high socio-ecoNomic and human rights impAct) project and includes the ethical guidelines for human enhancement research and development process. The project included a review of the state of the art, an analysis of legal and human rights requirements in and outside the EU, a survey of Research Ethics Committee approaches and codes, ethical assessment, surveys of societal acceptance and awareness, citizen panels and development of the ethical framework (23). The ethical framework consists of guidelines for human enhancement technologies (HET) and it proposed a research ethics framework as well as an international expert body to address the social, ethical and regulatory dimensions. The main difference made by the ethical framework was based on the aim to discern between: (1) projects in which human enhancement is a clear aim, either through research planned to facilitate human enhancement applications, or through the development of products or techniques projected for human enhancement, and (2) projects that have a possible “dual use” application, by which is meant that research and/or development is undertaken for therapeutic or other non-enhancement purposes, but the results of the project also have an explicit potential for human enhancement (22, 23).

This initiative is a valuable approach to set up the ethic framework for clinical research on human enhancement. In the next future global initiative should be promoted to harmonize research and prevent lack of transparency, establish ethics framework, and increase reproducibility.

The recent application of tools to edit the human genome with the intention of treating or preventing disease, in addition to some of the proposed applications of human genome editing, raised different ethical issues that have highlighted the need for robust oversight in this area. In December 2018, WHO established a global, multidisciplinary expert advisory committee (the Expert Advisory Committee on Developing Global Standards for Governance and Oversight of Human Genome Editing) to analyze the scientific, ethical, social and legal challenges related to human genome editing (somatic, germline and heritable). The recommendations of the Committee resulted in two publications on appropriate institutional, national, regional and global governance mechanisms for human genome editing, with particular attention to clinical trials. The main requirements are: (1) to guarantee that clinical studies using somatic human genome editing technologies are reviewed and approved by the appropriate research ethics committee before inclusion in the Registry of human genome editing clinical trials; (2) to develop an assessment mechanism to identify clinical trials using human genome editing technologies that may be of potential ethical concern; (3) to develop and review a set of international standards for clinical trials involving human genome editing for the clinical trials Registry; and (4) to support members of the scientific community to develop an additional basic and preclinical research registry (24).

However, the standards available could be still increased and improved. They should also define the borders of permissible practices, such as conducting research on non-viable embryos for advancing scientific knowledge and establishing ethical oversight committees to approve and monitor research projects involving viable embryos. The goal is to promote transparency in research practices, ensuring that ethical considerations are fully integrated into any gene editing study, and that gene editing research is conducted under strict guidelines to address the moral and social implications of altering germline cells, even if not intended for reproductive purposes.

### Registries, research, and traceability: building global infrastructure (s) for monitoring gene editing

International registries and global infrastructure should be created to track gene editing research and clinical applications and overcome the issue of transparency and traceability. This process foresees strict collaboration between academia, industry, governmental, non-governmental, and supranational organizations. The key actions are establishing mandatory registries that track all preclinical and clinical research on human enhancement technologies, including gene editing, developing standardized traceability systems for clinical practices involving enhancement to ensure accountability and transparency, and encouraging initiatives such as the “Genome Editing to Treat Human Diseases” (GenE-HumDi) network to foster global collaboration and standardize procedures in gene editing research (25). Additionally, platforms should be created for knowledge-sharing among researchers, institutions, and regulatory agencies to ensure consistent scientific practices across countries.

### Informed decisions

One of the main relevant issues is the lack of protocols to write comprehensive informed consent tailored to human enhancement technologies. These protocols should consider the exclusive challenges of enhancements that could involve epistemic gaps, where individuals cannot fully understand the long-term consequences, especially in the case of moral and cognitive enhancement. Individuals should be aware of potential risks, benefits, and unknowns of enhancements, with fully and accessible information about. Another recommendation to be included in the protocol is to provide access to multidisciplinary counseling with bioethicists, psychologists, and medical professionals, helping individuals navigate the ethical and personal implications of enhancement. Implement protections to restrict enhancements where fully informed consent may be compromised, ensuring that only those with a complete understanding of the risks are eligible for certain types of enhancement.

### Informed consent

Some scientists observed that it is impossible to obtain informed consent for germline therapy because the patients affected by the edits are the embryo and next generations (26). The counterargument is that parents already make many decisions that impact the future of their children. Researchers and bioethicists also worry about the risk of reaching a true informed consent from prospective parents if the risks of germline therapy are unknown (19).

Some enhancements including moral or cognitive modifications could raise an unbridgeable epistemic gap in key domains. These gaps could prove to be not merely unbridgeable due to a lack of information at a given moment, but radically unbridgeable, making someone in a non-enhanced state inherently unable to conceive of what it would be like to be enhanced in a particular way.

## Discussion

Today human enhancement is becoming increasingly integrated in our lives. Considering the different applications we analyzed, this new chapter of innovation asks for new and different ethics and health research questions. From our analysis emerged the need for awareness among people and the citizens of the possibility of enhancement, although this is not a systematic review on human enhancement regulation and ongoing research activity.

Enhancement could interest and involve different phases of life: conception, preventable disease, wellness, enhancement of brain capacity and treatment of serious and life-threatening diseases. There is also an urgent need to establish and share global rules for research among different Countries. Researchers should focus their attention also on methods for implementing this innovative process. This will require a lifelong learning journey considering previous Icarus’ effects, increasing knowledge and understanding the underlying process to bridge the gap between the starting point of the technology and human capacities and innovation. There is a lack of specialized programs to increase knowledge and skills for the next generation of stakeholders in health policy. These should be aimed at combining knowledge on new emerging technology or enhancement, legal frameworks, ethics and regulatory process. People that could be involved in this type of scholarship include physicians, lawyers, psychologists, bioethics experts.

Enhancement requires also deep discussion on social implications and the border between health and illness. The same applies to the border between prevention and enhancement of human capacity, where - for instance- gene therapy is the main example with its potential use for prevention of inherited genetic disease. Could enhancement find its application also on preventable and benignant diseases without risk of death or serious illness for patients? Up today there are no regulations aimed at defining the boarder of the application of the enhancement to these conditions.

The main point is what we are ready to accept regarding enhancement revolution: to aid people with disabilities and to improve the prognosis of serious diseases or to allow people to be much smarter, stronger and healthier than others.

## Data Availability

The original contributions presented in the study are included in the article/supplementary material, further inquiries can be directed to the corresponding author.
